# Orally active αvβ3 integrin inhibitor MK-0429 reduces melanoma metastasis

**DOI:** 10.3892/or.2015.3910

**Published:** 2015-04-09

**Authors:** MAUREEN PICKARSKI, ALEXA GLEASON, BOHUMIL BEDNAR, LE T. DUONG

**Affiliations:** 1 Bone Biology s, West Point, PA 19486, USA; 2 Imaging, Merck Research Laboratories, West Point, PA 19486, USA

**Keywords:** adhesion receptor, vitronectin receptor, malignant melanoma, lung metastasis

## Abstract

Melanoma remains one of the most aggressive types of cancer with a historically low survival rate. The αvβ3 integrin is involved in the progression of malignant melanoma. In the present study, the efficacy of MK-0429, a selective inhibitor of the αvβ3 integrin, was evaluated for its potential in the prevention of melanoma metastasis. Female B6D2F1 mice injected via the tail vein with murine B16F10 melanoma developed lung metastases within ~10 days. In the first experiment, the prevention of lung metastasis was assessed in the model treated with either vehicle, MK-0429 at 100 and 300 mg/kg orally twice daily or cyclophosphamide at 300 mg/kg, i.p. once daily. Study endpoints included determination of the study time period to achieve metastasis in lungs in this model, evaluation of the health effects on the study animals, the total number of lung colonies identified and lung tumor area. Unlike cyclophosphamide, the MK-0429 treatment did not lead to a significant weight reduction in mice. MK-0429 at 100 and 300 mg/kg reduced the number of metastatic tumor colonies by 64 and 57%, respectively, and the high dose also reduced the tumor area by 60% as compared to the vehicle. The second experiment employed B16F10 luciferase-expressing cells to examine the *de novo* progression of melanoma metastasis over 15 days with bioluminescent imaging of mice treated with MK-0429 at 300 mg/kg as compared to the vehicle. Tumor burden progressively advanced in the lungs of the B16F10-treated animals. However, MK-0429 reduced the progression of ventral and dorsal lung metastases by 22 and 38%, respectively, as compared to the vehicle, by study completion. Quantification of *ex vivo* tumor burden showed a 30–40% reduction in lung colonies by MK-0429. The two studies collectively demonstrated that MK-0429 was safe and efficacious in significantly decreasing melanoma metastasis in the lungs. The results emphasized the potential of MK-0429 as a novel, therapeutic agent for the prevention of metastatic melanoma.

## Introduction

Cutaneous melanoma remains the most aggressive type of cancer due to its invasiveness and propensity to metastasize (1). Malignant melanoma preferentially metastasizes to the lymph nodes, lungs and liver (2). While surgery and radiation therapy play a role in the palliation of the symptoms from local tumor growth, systemic therapy is the primary mode of treatment for metastatic melanoma, involving either single-agents or a combination of chemotherapy, immunotherapy or biochemical therapies. Combinations of these therapies may improve the response rate but do not extend survival and are associated with greater hematologic and multi-organ toxicity (3,4). As the incidence of melanoma continues to rise, significant unmet medical needs remain for novel, effective and safe therapies for the treatment of malignant melanoma.

The increased metastatic potential of melanoma has been associated with altered expression patterns of cell adhesion receptors including integrins (5,6). During melanoma progression from the benign melanocytic nervus to the metastatic melanoma, the melanoma lesion undergoes histopathologically distinct stages from the radial growth phase lesions to the vertical spreading into the adjacent papillary dermis-forming ‘vertical growth phase’ lesions (7,8). The tumor cells then enter the vasculature of the lymphatic system to invade and colonize distant target organs. Findings of previous studies suggest that integrin expression controls melanoma tumorigenicity by modulating cell migration (5,9), facilitating cell invasion and angiogenesis (10), while promoting tumor cell survival (11–13). Among the integrins associated with melanoma progression, the αvβ3 integrin, although not normally expressed on epidermal melanocytes or in most of the benign melanocytic nevi, has been found to be greatly increased as epidermal melanoma progresses to the vertical growth and invasive phases and is expressed in metastatic melanoma lesions (7).

Integrin αvβ3, a heterodimeric cell-surface adhesion receptor, specifically recognizes the arginine-glycine-aspartic (RGD) tripeptide sequence in a variety of extracellular matrix proteins, including vitronectin, osteopontin, fibrinogen, fibronectin, thrombospondin, von Willebrand factor and cryptic collagens (14,15). Notably, the integrin αvβ3 has been demonstrated to mediate osteoclastic bone resorption and endothelial neovascularization or angiogenesis. Significant upregulation of αvβ3 integrin expression is seen in tumoral endothelial cells, as well as in some tumor cells (9). Overexpression of αvβ3 by gene transfer in melanoma cell lines derived from the radial growth phase changed the properties of the cells to those of the vertical growth phase (16). Blocking antibodies to β3 integrin were reported to inhibit the migration, proliferation and metastasis of melanoma, as well as other tumor cells (17). Ligand engagement of αvβ3 integrin has been shown to induce melanoma cell growth by inhibiting apoptosis (11). Additionally, blocking of this integrin in human melanoma HT168M1 cells using an anti-β3 integrin monoclonal antibody resulted in the inhibition of lung colonization in an experimental metastasis assay (17). In that study, the anti-β3 integrin antibody recognized integrin αIIbβ3 and αvβ3 expressed in the human melanoma cell line, suggesting that the two integrins may participate in promoting tumor metastasis (17). Several RGD-disintegrins have also been shown to inhibit melanoma cell proliferation (18,19).

MK-0429 (L-000845704) is an orally active, potent and selective inhibitor of the integrin αvβ3 (20). As this inhibitor was originally developed for the treatment of osteoporosis, robust preclinical evidence demonstrated that MK-0429 potently inhibits binding of the ligand to the purified human integrin αvβ3 (IC_50_=0.08 nM) (20), and inhibits osteoclastic bone resorption (IC_50_=12.2±4.5 nM) *in vitro*. MK-0429 significantly reduces bone turnover and increases bone mass in ovariectomized rats and monkeys (20). In a randomized phase II trial, postmenopausal women with osteoporosis receiving MK-0429 at either 100 or 400 mg once daily, or 200 mg twice daily for 12 months showed a significant increase in bone mineral density at the spine and hip compared to those on placebo (21). In a small clinical study, men with hormone refractory prostate cancer and metastatic bone disease were administered MK-0429 at 200 and 1,600 mg orally, twice daily for four weeks (22). MK-0429 significantly decreased the bone resorption marker urinary N-telopeptide in these patients. In both clinical studies, MK-0429 was well tolerated and without serious adverse events.

In the present study, to gain better insight into the function of integrin αvβ3 in melanoma progression and metastasis, we first evaluated the efficacy of MK-0429 compared to cyclophosphamide in the prevention of metastatic melanoma progression using a model of murine B16F10 melanoma metastasis to the lungs. MK-0429 was then evaluated in a second experiment that employed B16F10 luciferase-expressing cells to examine the *de novo* progression of metastasis, wherein the treatment-associated effects on tumor progression in target tissues were evaluated with bioluminescent imaging *in vivo* and *ex vivo*.

## Materials and methods

### Synthesis of MK-0429

**Figure 1:**
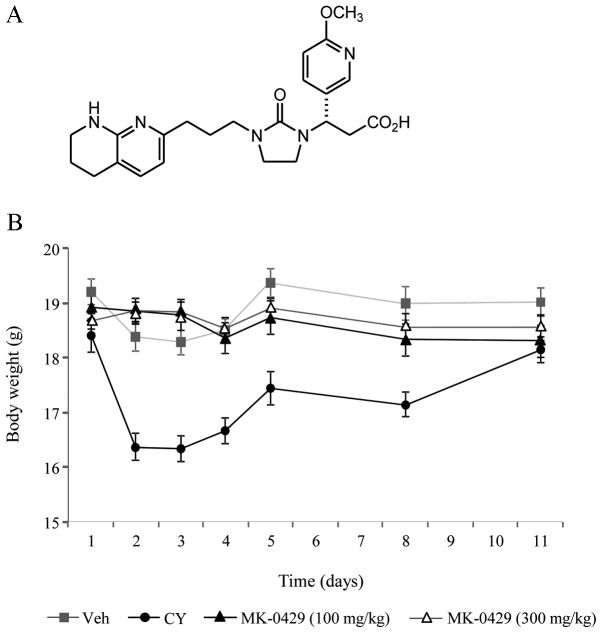
(A) Structure of MK-0429. (B) Treatment-associated effects on body weight of mice injected with B16F10 melanoma. CY-treated mice showed an initial loss of body weight, returning to baseline by day 11. Vehicle- or MK-0429-treated animals maintained their body weight throughout the study duration. Mean ± SEM; n=10–15/group. CY, cyclophosphamide; Veh, vehicle.

This compound is also known as L-000845704 (3(S)-(6-methoxypyridin-3-yl)-3-[2-oxo-3-[3-(5,6,7,8-tetrahydro-[1,8]-naphthyridin-2-yl) propyl] imidazolidin-1-yl] propionic acid). The synthesis and structure of MK-0429 (Fig. 1a) were as previously described (20).

### Integrin-mediated binding assays

Human embryonic kidney 293 (HeK293) cells were stably co-transfected with human integrin subunits αv and β3 to establish the HeK293-αvβ3 cell line as previously described (23). Purification of these integrins and assay conditions were performed as previously described (20). Briefly, the affinity of ^3^H-MK-0429 for various integrins was determined by binding assays using the purified receptors.

### Cell adhesion assays

HeK293 cells overexpressing human integrins αvβ3, αvβ5, αIIbβ3 or α5β1 were established as previously described (23,24). The cells (25×10^3^ cells/well) were added to microtiter wells that were coated with vitronectin (αvβ3 and αvβ5), fibrinogen (αIIbβ3) or fibronectin (α5β1) and were allowed to attach for 2 h at 37°c in a humidified incubator in the absence or presence of increasing concentrations of MK-0429. The non-attached cells were gently washed away. The attached cells were quantified by colorimetric detection of hexosaminidase enzymatic activity (25) in a Vmax micro-plate reader (Molecular Devices, Menlo Park, CA, USA). The number of attached cells was quantified using a standard curve for each cell line assayed and expressed as a mean value of triplicate samples.

### B16F10 melanoma cell lines

The B16F10 murine melanoma cell line, was obtained from the American Type Culture Collection (ATCC; Manassas, VA, USA). The cells were routinely cultured in high glucose Dulbecco’s modified eagle’s medium (DMEM) supplemented with 10% fetal bovine serum (FBS) and 1% penicillin/streptomycin (Life Technologies, Carlsbad, CA, USA) at 37°C in a humidified 5% CO_2_ incubator. Stable expression of luciferase was established in the B16F10 melanoma cells through Geneticin (G418) selection. Briefly, B16F10 cells were transfected with pcDNA3.1 and pGL3 plasmid using Lipofectamine 2000 (Life Technologies, Carlsbad, CA, USA) according to the manufacturer’s instructions. The cells were then selected in 1,250 *μ*g/ml G418 (Life Technologies) for two weeks and individual colonies were isolated, expanded and maintained in G418. Luciferase expression in these clones was confirmed by flow cytometry and bioluminescent imaging.

### Reverse transcription and TaqMan

The total RNA was extracted using an RNeasy kit (Qiagen, Valencia, CA, USA) according to the manufacturer’s instructions. Reverse transcription and real-time PCR were performed as previously described (26). Primer/probe pairs for real-time PCR were as follows (Life Technologies): ITGAV forward, 5′-CGGGTCCCGAGGGAAGT-3′ and reverse, 5′-GGGTCGTGTTCGCTTTGG-3′), and fluorogenic probe, 5′-TCGAGCCCAGCACGTCCTCCA-3′; ITGB3 forward, 5′-GATGCTTACGGGAAAATCCG-3′ and reverse, 5′-TTGAAGGACAGTGACAGCTCTCC-3′, and fluorogenic probe, 5′-CTAAAGTGGAGCTGGAAGTACGTGACCTGC-3′); and ITGB5 forward, 5′-GGTTTCGGGTCTTTTGTTGACA-3′ and reverse, 5′-GGAATAACTTGTAACCAATACACGGA-3′, and fluorogenic probe, 5′-TCTCTCCTTTCTCCTACACGGCACCGA-3′.

### Development of lung metastasis in the murine B16F10 melanoma model

The in-life portion of the present study was conducted at the Piedmont Research Center (Morrisville, NC, USA). The Piedmont Research Center complies with the recommendations of the Guide for Care and Use of Laboratory Animals and is accredited by AAALAC International, which assures compliance with accepted standards for the care and use of laboratory animals. In total, 55 6-week-old B6D2F1 hybrid female mice received 1.5×10^5^ B16F10 melanoma cells by intravenous (i.v.) tail vein injection. The animals received either saline, MK-0429 at 100 or 300 mg/kg, p.o., twice daily (b.i.d.) or cyclophosphamide, 300 mg/kg, i.p., once daily (q.d.), 1 day post-tumor injection, n=10–15/group. The body weight was recorded daily to determine whether treatment affected the health of the animals. Necropsy was performed when the number of lung colonies reached 100 metastases/lung counted from a separate set of control mice, ~2 weeks after study initiation (27,28). Lungs were dissected with minimal bronchus attached. Melanoma colonies on the surface of all lung regions were counted.

Histological analysis of the mouse lungs was performed. The mouse lungs were fixed in 10% formalin. After being embedded, the samples were sectioned beginning at ~1 mm into the tissue along the frontal plane. The sections were stained with hematoxylin and eosin (H&E) followed by imaging and tumor area quantification using ImagePro software.

### Non-invasive bioluminescent imaging of de novo lung metastasis in the murine B16F10 melanoma model

The in-life portion of the present study was conducted at Merck Research Laboratories (Rahway, NJ, USA). Animal procedures were in accordance with the national guidelines and were approved by the Institutional Animal Care and Use Committee (IACUC) at Merck.

Twenty 8-week-old (nu/nu) female mice, were injected with 2.5×10^5^ B16F10-luc melanoma cells by i.v. tail vein injection. The animals received saline or MK-0429, 300 mg/kg, p.o., b.i.d., 1 day post-tumor injection, n=10/group. Bioluminescence imaging was performed using the Xenogen IVIS 200 (Perkin-Elmer, Waltham, MA, USA) twice/week until the study end and images were quantified using Living image software. The mice were anesthetized with 2.5% isoflurane prior to imaging and then a 90 mg/kg dose of D-luciferin, i.p. (Perkin-Elmer). Exposure time was adjusted to avoid pixel saturation. Default settings for bioluminescent scanning were used for scanning at each time-point. A total bioluminescent flux (photons/second) was calculated for each region of interest (ROI). Square ROIs were placed around the head, lung and abdomen of each animal for each scan followed by image analysis of head, lungs and abdomen, on dorsal and ventral planes.

The animals were sacrificed by carbon dioxide asphyxiation. Following necropsy, *ex vivo* bioluminescent imaging of the lungs was performed by Xenogen IVIS 200. Default bioluminescent settings of Living Image were used with exposure times manually adjusted to avoid saturation. ROIs were placed on the 2D bioluminescent image to encompass the entire lung tissues. Melanoma colonies on the surface of the lung regions were counted.

### Statistical analysis

Data are presented as mean ± SEM and were analyzed with GraphPad Prism 6 software (San Diego, CA, USA). Study endpoints were tested for Gaussian distribution. Statistical analysis was performed by the unpaired Student’s t-test or the one-way ANOVA followed by the Tukey’s multiple comparison test. The histological quantification of the tumor area was analyzed using StatView, followed by the Fisher’s PLSD test. P<0.05 was considered to indicate a statistically significant result.

## Results

### Potency and safety profile of MK-0429 and integrin expression profile of B16F10 melanoma

The structure of MK-0429 (Fig. 1a) was previously described (20). MK-0429 binds with high affinity to the purified human αvβ3 integrin. The equilibrium dissociation constants (Kds) of ^3^H-MK-0429 in binding to the purified human, murine and rat αvβ3 integrin are 0.33±0.04, 0.56±0.07 and 1.23±0.11 nM, respectively. This inhibitor blocks the adhesion of HeK293-αvβ3 cells to vitronectin with an IC_50_ of 0.58±0.30 nM. MK-0429 is ~100-fold less potent in blocking the adhesion of HeK293 overexpressing the closely related αvβ5 integrin to vitro-nectin, and >1,000-fold less active in blocking adhesion functions mediated by integrins αIIbβ3 or α5β1 to fibrinogen or fibronectin, respectively.

The mRNA expression levels of integrin subunits were determined for the highly metastatic B16F10 cell line. Integrin αv was the predominant subunit, demonstrating a mRNA expression ~8-fold greater than that of the β5 subunit. The β3 subunit was detectable at the cycle threshold values near 40 (data not shown), consistent with previous reports from the FACS analysis (29). Having established detectable expression of the subunits of the vitronectin receptors in the melanoma cell line, we then investigated MK-0429 as a potential therapeutic for the treatment of melanoma.

### Effects of MK-0429 on body weight of mice injected with melanoma cells

MK-0429 has been demonstrated to be well tolerated and efficacious in preclinical and clinical studies of osteoporosis (21,22). In the present study, we evaluated its effect on body weight compared to cyclophosphamide in mice employing a B16F10 murine melanoma model in the prevention mode. Animals received tail-vein injection of B16F10 melanoma cells followed by treatment with vehicle (Veh), MK-0429 (at 100 and 300 mg/kg, p.o., b.i.d.) or cyclophosphamide (CY; 300 mg/kg, i.p., q.d.) one day after cell inoculation. To validate the utility of the model, metastatic lung nodule development was monitored in a separate cohort, with ~100 metastatic lung colonies developing within two weeks of B16F10 cell inoculation and this time period was defined as the operative study duration (data not shown). Veh- and MK-0429-treated animals showed no significant weight loss over the study duration (Fig. 1B). By contrast, the CY-treated animals experienced a rapid loss of weight in the first four days of the study, losing ~9–11% of their total body weight. This was followed by a return towards the baseline weight levels by the end of the study (Fig. 1B).

### MK-0429 reduces metastatic tumor colony formation and area in the lungs

**Figure 2:**
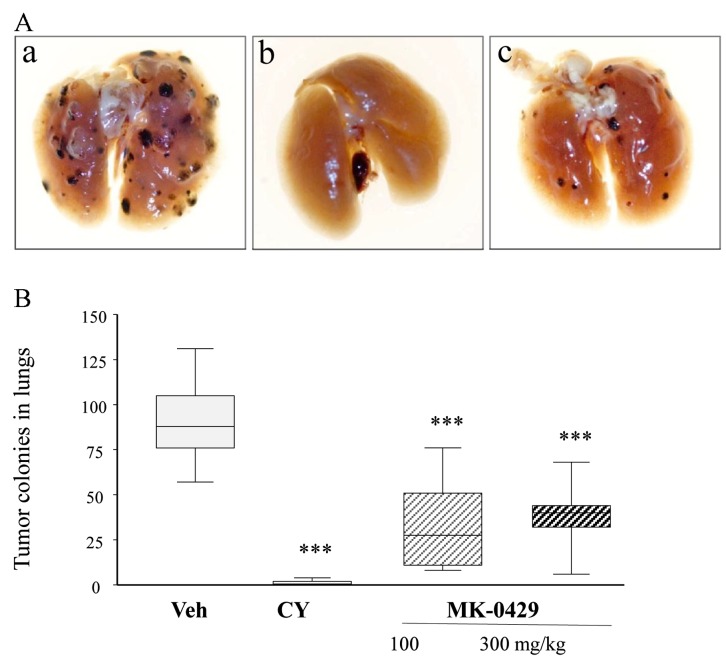
MK-0429 significantly reduces the total number of melanoma lung colonies in the B16F10 melanoma prevention model. (A) Gross appearance of pulmonary metastasis clearly visible in vehicle-treated animals (a). Notably fewer colonies are present with (b) cyclophosphamide and (c) MK-0429 treatment. (B) Quantitative analysis of the total number of tumor colonies in all lung regions demonstrates that MK-0429 reduced the tumor colony number by 64 and 57%, at 100 and 300 mg/kg, respectively, as compared to the vehicle control. Cyclophosphamide eradicated almost all the tumor colonies, 99% as compared to vehicle control. ^***^p<0.001 vs. Veh. Data are presented as mean ± SEM; n=10–15/group. Veh, vehicle.

The extent of lung metastasis and the effect of drug treatment were assessed by examining the lung surfaces for observable melanoma tumor colony formation. Following termination of the study, lung surfaces were visually inspected and the total number of *ex vivo* melanoma colonies on the lung surface was counted. The gross appearance of representative *ex vivo* lung sets revealed clearly visible metastatic melanoma colonies (dark spots) in Veh-treated animals and notably fewer observable colonies following CY or MK-0429 treatment (Fig. 2A). CY and MK-0429 effectively reduced the number of tumor colonies on the entire lung surface (Fig. 2B). The mean number of the metastatic melanoma nodules was 90±5 in the Veh-treated controls. Cyclophosphamide treatment eradicated almost all the tumor colonies, 99% as compared to that in the Veh control (p<0.001). MK-0429 treatment also decreased the total tumor colony number to 33±6 and 39±9, at 100 and 300 mg/kg, respectively, (p<0.001 vs. Veh) thus reducing tumor burden by 64 and 57%.

**Figure 3:**
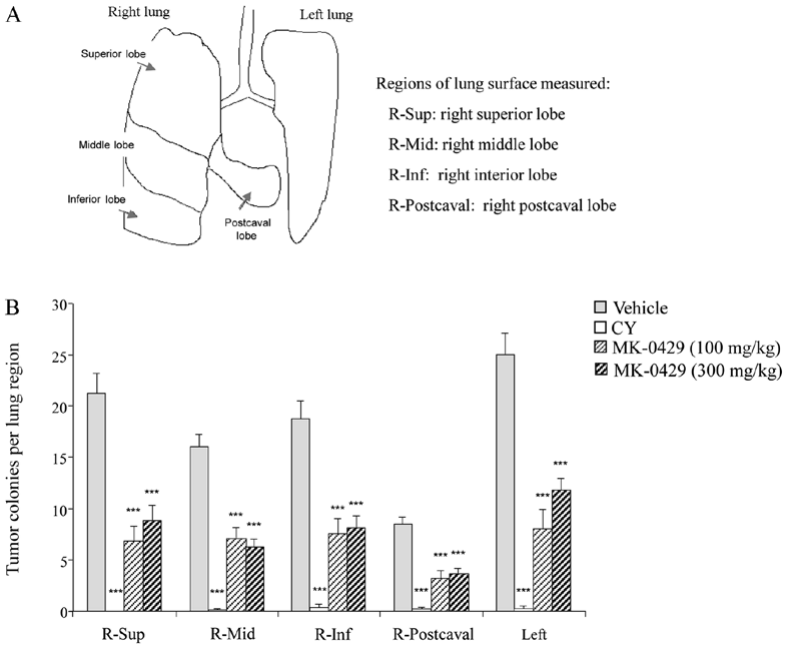
Evaluation of lung metastasis on each lung region in the B16F10 melanoma prevention model. (A) A diagram indicating each of the five lung regions scored for metastatic lung colonies. (B) Cyclophosphamide and MK-0429 at 100 and 300 mg/kg were effective in significantly reducing the tumor burden in each lung region. ^***^p<0.001 vs. Veh. Data are presented as mean ± SEM; n=10–15/group. Veh, vehicle.

The lung was subdivided into five separate compartments to determine whether particular surface areas were preferentially affected by metastasis or drug treatment (Fig. 3A). Tumor colonies were manually counted within each lung region. In the Veh-treated animals, the scope of tumor colonies across the lung surfaces averaged 9–25 tumor colonies/individual lung surface (Fig. 3B). The largest lung surface, the left lung, contained the highest number of melanoma colonies (25±8), whereas the smallest lung surface, the post caval lobe, showed the least tumor colony formation (9±1). Metastatic melanoma burden was significantly reduced within each lung compartment with drug treatment. Consistent with the results from the entire lung surface, CY treatment markedly reduced tumor formation in each of the five lung regions (p<0.001 vs. Veh). MK-0429 at the two doses significantly reduced melanoma colonies in all the lung regions by 53–68% indicating no regional effect of treatment (p<0.001 vs. Veh) (Fig. 3B).

**Figure 4:**
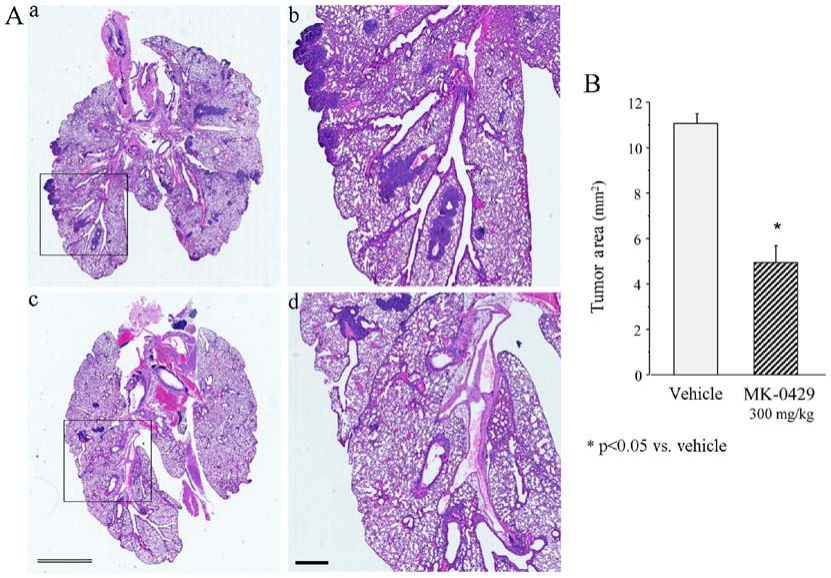
MK-0429 reduces tumor area in lungs. (A) Representative images of H&E-stained lung sections harvested from (a and b) vehicle- and (c and d) MK-0429 300 mg/kg, b.i.d.-treated animals. High magnification of the rectangular regions in a and c (bar, 5 mm) are shown as b and d (bar, 1 mm), respectively. Metastatic melanoma colonies appear as dark purple regions within the lung tissues. (B) Quantitative analysis of the tumor area shows that MK-0429 significantly reduced tumor area by 60% as compared to the vehicle control. ^*^p<0.01 vs. Veh. Data are presented as mean ± SEM; n=5/group. H&E, hematoxylin and eosin; Veh, vehicle.

The presence of metastatic lesions in the lungs was confirmed by histological analysis of the sections from the Veh- and MK-0429 (300 mg/kg)-treated animals. Fig. 4Aa and b shows a typical H&E-stained section of Veh-treated lungs containing melanoma colonies. However, fewer colonies were present in the MK-0429-treated lungs (Fig. 4Ac and d). Veh-treated animals showed a total tumor area of 11.1±0.4 mm^2^. The total number and area of the melanoma colonies was significantly reduced following treatment with MK-0429 to 5.0±0.7 mm^2^ (p<0.01 vs. Veh) (Fig. 4B). Treatment with MK-0429 decreased the tumor area by 60% compared to that in the Veh controls, consistent with the treatment-associated reduction of the number of melanoma colonies.

### MK-0429 reduces the de novo progression of lung metastases

The second experiment focused on the *de novo* progression patterns of metastatic spread and the resulting effect of drug treatment on melanoma metastasis in mice by utilizing non-invasive imaging and B16F10 melanoma cells stably transfected with luciferase. *In vitro* bioluminescent imaging confirmed the expression levels of luciferase for B16F10 clones selected for *in vivo* studies. Additionally, real-time PCR demonstrated integrin subunit mRNA levels consistent with the parental cell line (data not shown). Athymic mice were injected with 2.5×10^5^ B16F10-luc cells. One-day post injection, the animals were orally administered either Veh or MK-0429, 300 mg/kg, b.i.d. for two weeks. The animals were imaged for bioluminescence on day 0, 3, 8, 11 and 15 to determine the time required to develop measurable lung metastases and the extent of metastatic spread within other organs. Tumor progression was monitored and quantified by image analysis in the head, lungs and abdomen.

**Figure 5:**
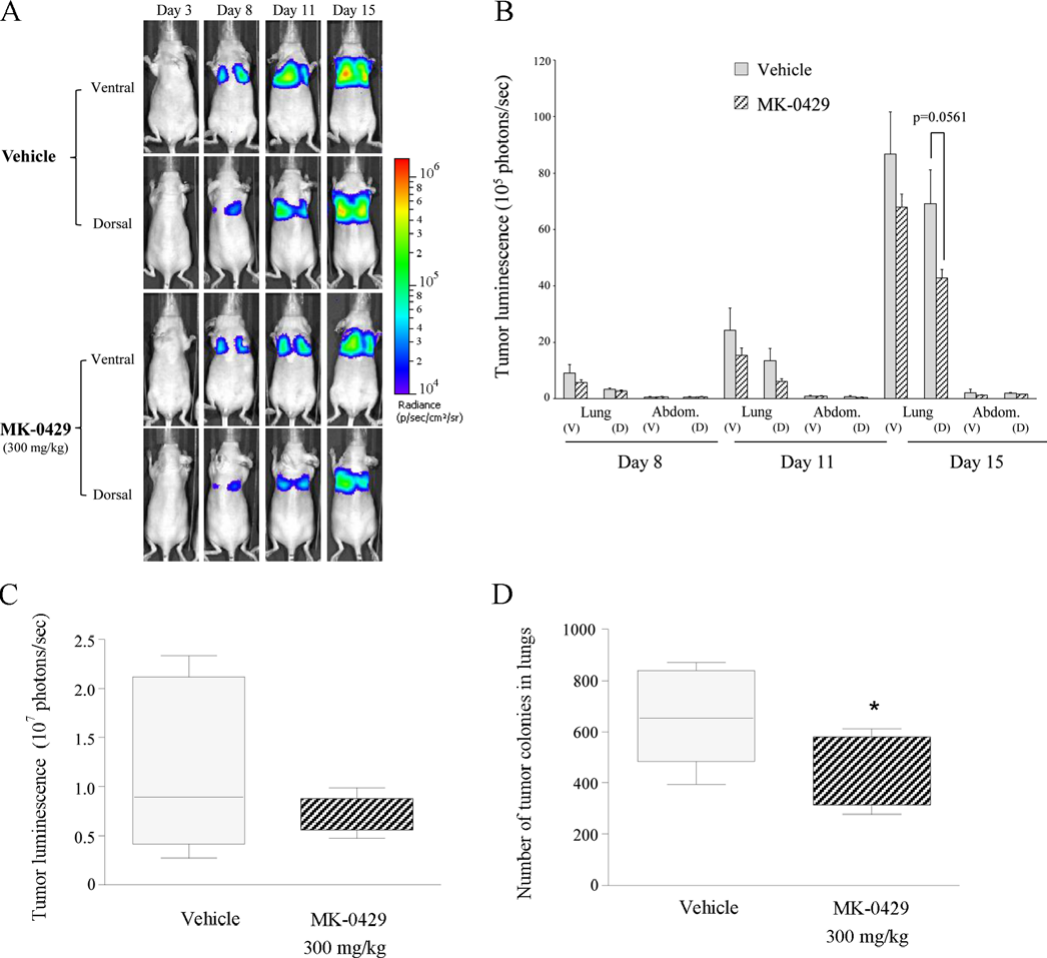
Effect of MK-0429 on tumor growth and formation of de novo metastasis. (A) Representative luminescent images demonstrating time-dependent progression of metastasis in vehicle-treated animals and reduced tumor burden in MK-0429-treated animals. (B) Tumor burden represented by luminescence in the lungs and abdomen (a and b) on day 8, 11 and 15, ventral (v) and dorsal (d). (C) Tumor burden represented by luminescence in the lungs *ex vivo* was reduced by MK-0429 treatment. (D) The number of tumor colonies is significantly reduced by MK-0429 treatment. p<0.05 vs. Veh. Mean ± SEM; n=10/group. Veh, vehicle.

Fig. 5A shows a progressive increase in tumor signal from day 3 through day 15 in the study animals. Representative bioluminescent images show that tumor progression was negligible in the abdomen and head throughout the study time course. However, melanoma burden gradually increased in the lungs in the dorsal and ventral planes in the Veh-treated animals (Fig. 5A). Tumor burden advanced less aggressively in MK-0429-treated animals. Quantification of tumor burden demonstrated a time-dependent increase in lung metastases in the Veh controls, whereas the abdomen and head showed little detectable signal above the background in the Veh- and drug-treated animals (Fig. 5B). MK-0429 300 mg/kg on day 8, 11 and 15, reduced melanoma burden in the ventral lung by 38, 37 and 21%, respectively. Similarly, MK-0429 reduced tumor metastases in the dorsal lung by 15, 54 and 38% (p=0.0561 vs. Veh), respectively, as compared to the Veh controls (Fig. 5B). Lung metastasis was confirmed by *ex vivo* bioluminescent analysis. Veh-treated lungs demonstrated a tumor burden of 118.1±27.1×10^5^ photons/sec. MK-0429 decreased metastasis in the lungs by ~40%, reducing melanoma burden to 69.9±5.9×10^5^ photons/sec. This difference was not statistically significant (Fig. 5c). However, the number of visible metastatic colonies counted on the *ex vivo* lung surface was significantly reduced by 32% by MK-0429 treatment twice daily for two weeks (p<0.05 vs. Veh), consistent with the percentage reduction of tumor burden observed by bioluminescent imaging (Fig. 5D). Thus, evaluation of melanoma burden at study termination by *in vivo* and *ex vivo* bioluminescent imaging, as well as by manual melanoma colony count, indicated an ~30–40% reduction in lung metastasis following treatment with MK-0429 300 mg/kg administered twice daily as compared to the Veh-treated controls.

## Discussion

During melanoma progression from the benign melanocytic nevus to the metastatic melanoma, the melanoma lesion undergoes several clinically and histopathologically distinct stages (7,8). Initially, the radial growth phase lesions are limited to the epidermis and are essentially benign. Then, the cells begin to spread vertically into the adjacent papillary dermis, while they continue to invade the adjacent reticular dermis, subcutaneous fat and eventually enter the lymphatics and vascular circulation. Degradation of the basement membranes and vascularized structures around malignant melanoma are also crucial to local invasion and hematogenous metastases of melanoma (8). Expression of αvβ3 integrin, previously found to be involved in the regulation of cell growth, motility and invasion (30,31), coincides with progression of the invasive phase of melanocytes to the vertical growth phase of metastatic melanoma (32,33). Furthermore, αvβ3 integrin is involved in angiogenesis as well as in several processes of melanoma metastasis by promoting cell proliferation, attachment, transendothelial migration and invasion through an interaction with MMP-2 to support cell intravasation, extravasation and target organ colonization (34,35). Collectively, the above mentioned data raise the possibility that blocking the functions of αvβ3 integrin prevents the early stages of melanoma metastasis.

The focus of this preclinical study was to evaluate the role of this adhesion receptor αvβ3 in melanoma in target organ colonization. MK-0429, a known selective inhibitor of human αvβ3 integrin (20), also blocks adhesion of the HEK293-αvβ5 cells, albeit at ~100-fold lower efficacy compared to that of αvβ3 integrin (23). The selectivity profile of MK-0429 was further demonstrated by its lack of potency in inhibition in a panel of adhesion assays, including the attachment of HEK293 cells overexpressing the αIIbβ3 or α5β1 integrin to fibrinogen or fibronectin, respectively (25). Although the expression levels of αvβ5 integrin appear to be higher than that of αvβ3 integrin in B16F10 cells, the selectivity profile of MK-0429 supported the predominant role of αvβ3 integrin in the colonization and growth of this murine melanoma in the lungs.

While high expectations for the mechanism of αvβ3 integrin in mediating tumor angiogenesis, growth and invasion have derived from the significant body of positive preclinical and early clinical findings (36), blocking antibody or small molecular weight inhibitors led to mostly negative results from various phase II and III trials in pancreatic, prostate, head and neck cancers, glioblastoma and melanoma (9,37–40). Etaracizumab is a monoclonal antibody against αvβ3 that was evaluated at 8 mg/kg once weekly, i.v., and administered for two cycles of a 3-week infusion in 112 patients with stage IV metastatic melanoma in the presence and absence of dacarbazine for a year (41). The median survival rate was not different in patients treated with etaracizumab alone or in combination with dacarbazine, suggesting etaracizumab treatment was unlikely to result in clinically meaningful improvement over dacarbazine alone (41). Cilengitide (EMD 121974), a dual inhibitor of αvβ3 and αvβ5 integrins, was administered intravenously twice weekly in a small number of patients (n=12–14) with stage IV or unresectable stage III metastatic melanoma to assess the clinical efficacy of cilengitide in the progression-free survival rate at eight weeks (38). Cilengitide demonstrated minimal clinical efficacy as a single-agent therapy for advanced melanoma (38). Despite the implications of the role of αvβ3 integrin involvement in the early stages of melanoma metastasis, the aim of these previous studies was clearly geared towards testing efficacy of αvβ3 inhibitors in patients in later stages of advanced melanoma.

Other pitfalls of the early trials may explain the lack of efficacy of anti-αvβ3 integrin in the melanoma population. Targeting of αvβ3 in tumor tissues is complex due to the dose-dependent opposing effects of the inhibitor: low doses of RGD-peptides have been reported to stimulate VEGF-blood vessel growth and tumor angiogenesis (42), in contrast with inhibition at higher doses (36). Alternative adhesion receptors, including αvβ5 or α5β1 integrins, may also compensate for tumor angiogenesis (36). Metabolic imaging and tissue analysis suggested that etaracizumab or cilengitide reached the target tissues. However, little is known regarding the effect of these drugs on tumor vasculature or invasiveness in patients. Moreover, etaracizumab and cilengitide have relatively short half-lives of approximately a few days and 2–4 h, respectively. Thus, unfavorable pharmacokinetics and non-continuous dosing regimens of these drugs may partly explain the negative results. Intravenous administration of these drugs, together with the infrequency of dosing intervals, may be suboptimal to achieve appropriate anti-angiogenic or anti-invasion pressure, which may require persistent long-term therapy for effectiveness.

An issue that is to be considered is whether the mechanism of blocking αvβ3 integrin should be re-evaluated with an orally active agent with a proven excellent safety profile in the clinic, particularly in the context of early intervention of the disease course, such as preventing systemic spread. MK-0429 may be suitable for this approach. Although safety and efficacy of MK-0429 were clearly demonstrated in animal models (20) and in postmenopausal osteoporotic women for two years (21), the safety profile of this compound has also been recently tested at the maximally tolerated oral dose of 1,600 mg twice daily for four weeks in men with hormone refractory prostate cancer and metastatic bone disease (22). Markedly, MK-0429 was well tolerated at this high dose.

At present, there are three classes of therapies approved for melanoma, the alkylating agents such as dacarbazine, the targeted therapies such as the selective inhibitor of BRAF V600E, and immunotherapeutic agents including anti-CTLA-4 antibody and more recently anti-PD-1 antibody (43). Dacarbazine remains the gold standard in chemotherapy, while combining dacarbazine with new pharmacological or immunotherapeutic agents is currently under evaluation in order to achieve better clinical responses in patients with advanced melanoma. The development of targeted- and immuno-therapies has changed the paradigm for treatment of patients with advanced melanoma, although not all patients respond to these therapies. The lack of predictive biomarkers of response is probably the major weakness of these novel therapies. The recent development of MK-0429-based imaging markers may be useful to monitor melanoma responses to therapies (25). Furthermore, for future therapy of metastatic melanoma, the challenge is to expand and combine the current therapies to prevent systemic metastasis at the earlier stage of the disease. In the present study, we have provided new insight into the application of a novel small molecule integrin inhibitor with an excellent safety profile as a therapeutic agent for the early prevention of metastatic melanoma. Combination of the αvβ3 integrin inhibitor with the current standard of care may improve pharmacologic management and clinical outcomes for patients with metastatic melanoma.

## References

[1] Balch CM, Soong SJ, Gershenwald JE, Thompson JF, Reintgen DS, Cascinelli N, Urist M, McMasters KM, Ross MI, Kirkwood JM (2001). Prognostic factors analysis of 17,600 melanoma patients: Validation of the American Joint Committee on Cancer melanoma staging system. J Clin Oncol.

[2] Cummins DL, Cummins JM, Pantle H, Silverman MA, Leonard AL, Chanmugam A (2006). Cutaneous malignant melanoma. Mayo Clin Proc.

[3] Culos KA, Cuellar S (2013). Novel targets in the treatment of advanced melanoma: New first-line treatment options. Ann Pharmacother.

[4] Ma C, Armstrong AW (2014). Severe adverse events from the treatment of advanced melanoma: A systematic review of severe side effects associated with ipilimumab, vemurafenib, interferon alfa-2b, dacarbazine and interleukin-2. J Dermatolog Treat.

[5] Kuphal S, Bauer R, Bosserhoff AK (2005). Integrin signaling in malignant melanoma. Cancer Metastasis Rev.

[6] Marshall JF, Rutherford DC, Happerfield L, Hanby A, McCartney AC, Newton-Bishop J, Hart IR (1998). Comparative analysis of integrins in vitro and in vivo in uveal and cutaneous melanomas. Br J Cancer.

[7] Nip J, Brodt P (1995). The role of the integrin vitronectin receptor, alpha v beta 3 in melanoma metastasis. Cancer Metastasis Rev.

[8] Johnson JP (1999). Cell adhesion molecules in the development and progression of malignant melanoma. Cancer Metastasis Rev.

[9] Desgrosellier JS, Cheresh DA (2010). Integrins in cancer: Biological implications and therapeutic opportunities. Nat Rev Cancer.

[10] Jin H, Varner J (2004). Integrins: Roles in cancer development and as treatment targets. Br J Cancer.

[11] Petitclerc E, Strömblad S, von Schalscha TL, Mitjans F, Piulats J, Montgomery AM, Cheresh DA, Brooks PC (1999). Integrin α_v_β_3_ promotes M21 melanoma growth in human skin by regulating tumor cell survival. Cancer Res.

[12] Smith SD, Enge M, Bao W, Thullberg M, Costa TD, Olofsson H, Gashi B, Selivanova G, Strömblad S (2012). Protein kinase Cα (PKCα) regulates p53 localization and melanoma cell survival downstream of integrin αv in three-dimensional collagen and in vivo. J Biol Chem.

[13] Bao W, Strömblad S (2004). Integrin alphav-mediated inactivation of p53 controls a MEK1-dependent melanoma cell survival pathway in three-dimensional collagen. J Cell Biol.

[14] Horton MA (1997). The alpha v beta 3 integrin ‘vitronectin receptor’. Int J Biochem Cell Biol.

[15] Eble (1997). Integrin-Ligand Interaction.

[16] Hsu MY, Shih DT, Meier FE, Van Belle P, Hsu JY, Elder DE, Buck CA, Herlyn M (1998). Adenoviral gene transfer of beta3 integrin subunit induces conversion from radial to vertical growth phase in primary human melanoma. Am J Pathol.

[17] Trikha M, Zhou Z, Timar J, Raso E, Kennel M, Emmell E, Nakada MT (2002). Multiple roles for platelet GPIIb/IIIa and alphavbeta3 integrins in tumor growth, angiogenesis, and metastasis. Cancer Res.

[18] Oliva IB, Coelho RM, Barcellos GG, Saldanha-Gama R, Wermelinger LS, Marcinkiewicz C, Benedeta Zingali R, Barja-Fidalgo C (2007). Effect of RGD-disintegrins on melanoma cell growth and metastasis: Involvement of the actin cytoskeleton, FAK and c-Fos. Toxicon.

[19] Kang IC, Kim DS, Jang Y, Chung KH (2000). Suppressive mechanism of salmosin, a novel disintegrin in B16 melanoma cell metastasis. Biochem Biophys Res Commun.

[20] Hutchinson JH, Halczenko W, Brashear KM, Breslin MJ, Coleman PJ, Duong T, Fernandez-Metzler C, Gentile MA, Fisher JE, Hartman GD (2003). Nonpeptide alphavbeta3 antagonists. 8. In vitro and in vivo evaluation of a potent alphavbeta3 antagonist for the prevention and treatment of osteoporosis. J Med Chem.

[21] Murphy MG, Cerchio K, Stoch SA, Gottesdiener K, Wu M, Recker R, L-000845704 Study Group (2005). Effect of L-000845704, an alphaVbeta3 integrin antagonist, on markers of bone turnover and bone mineral density in postmenopausal osteoporotic women. J Clin Endocrinol Metab.

[22] Rosenthal MA, Davidson P, Rolland F, Campone M, Xue L, Han TH, Mehta A, Berd Y, He W, Lombardi A (2010). Evaluation of the safety, pharmacokinetics and treatment effects of an α_v_β_3_ integrin inhibitor on bone turnover and disease activity in men with hormone-refractory prostate cancer and bone metastases. Asia Pac J Clin Oncol.

[23] Simon KO, Nutt EM, Abraham DG, Rodan GA, Duong LT (1997). The alphavbeta3 integrin regulates alpha5beta1-mediated cell migration toward fibronectin. J Biol Chem.

[24] Abraham DG, Nutt EM, Bednar RA, Bednar B, Gould RJ, Duong LT (1997). Arginine-glycine-aspartic acid mimics can identify a transitional activation state of recombinant alphaIIb beta3 in human embryonic kidney 293 cells. Mol Pharmacol.

[25] Kossodo S, Pickarski M, Lin SA, Gleason A, Gaspar R, Buono C, Ho G, Blusztajn A, Cuneo G, Zhang J (2010). Dual in vivo quantification of integrin-targeted and protease-activated agents in cancer using fluorescence molecular tomography (FMT). Mol Imaging Biol.

[26] Pickarski M, Hayami T, Zhuo Y, Duong T (2011). Molecular changes in articular cartilage and subchondral bone in the rat anterior cruciate ligament transection and meniscectomized models of osteoarthritis. BMC Musculoskelet Disord.

[27] Fidler IJ (1975). Biological behavior of malignant melanoma cells correlated to their survival in vivo. Cancer Res.

[28] Fidler IJ, Kripke ML (1977). Metastasis results from preexisting variant cells within a malignant tumor. Science.

[29] Cowden Dahl KD, Robertson SE, Weaver VM, Simon MC (2005). Hypoxia-inducible factor regulates alphavbeta3 integrin cell surface expression. Mol Biol Cell.

[30] Barczyk M, Carracedo S, Gullberg D (2010). Integrins. Cell Tissue Res.

[31] Giancotti FG, Ruoslahti E (1999). Integrin signaling. Science.

[32] Van Belle PA, Elenitsas R, Satyamoorthy K, Wolfe JT, Guerry D, Schuchter L, Van Belle TJ, Albelda S, Tahin P, Herlyn M (1999). Progression-related expression of beta3 integrin in melanomas and nevi. Hum Pathol.

[33] Albelda SM, Mette SA, Elder DE, Stewart R, Damjanovich L, Herlyn M, Buck CA (1990). Integrin distribution in malignant melanoma: Association of the beta 3 subunit with tumor progres­sion. Cancer Res.

[34] Brooks PC (1996). Role of integrins in angiogenesis. Eur J Cancer.

[35] Brooks PC, Strömblad S, Sanders LC, von Schalscha TL, Aimes RT, Stetler-Stevenson WG, Quigley JP, Cheresh DA (1996). Localization of matrix metalloproteinase MMP-2 to the surface of invasive cells by interaction with integrin alpha v beta 3. Cell.

[36] Danen EHJ (2013). Integrin signaling as a cancer drug target. ISRN Cell Biol.

[37] Reardon DA, Fink KL, Mikkelsen T, Cloughesy TF, O’Neill A, Plotkin S, Glantz M, Ravin P, Raizer JJ, Rich KM (2008). Randomized phase II study of cilengitide, an integrin-targeting arginine-glycine-aspartic acid peptide, in recurrent glioblastoma multiforme. J Clin Oncol.

[38] Kim KB, Prieto V, Joseph RW, Diwan AH, Gallick GE, Papadopoulos NE, Bedikian AY, Camacho LH, Hwu P, Ng CS (2012). A randomized phase II study of cilengitide (EMD 121974) in patients with metastatic melanoma. Melanoma Res.

[39] Gutheil JC, Campbell TN, Pierce PR, Watkins JD, Huse WD, Bodkin DJ, Cheresh DA (2000). Targeted antiangiogenic therapy for cancer using Vitaxin: A humanized monoclonal antibody to the integrin alphavbeta3. Clin Cancer Res.

[40] Mcneel DG, Eickhoff J, Lee FT, King DM, Alberti D, Thomas JP, Friedl A, Kolesar J, Marnocha R, Volkman J (2005). Phase I trial of a monoclonal antibody specific for alphavbeta3 integrin (MEDI-522) in patients with advanced malignancies, including an assessment of effect on tumor perfusion. Clin Cancer Res.

[41] Hersey P, Sosman J, O’Day S, Richards J, Bedikian A, Gonzalez R, Sharfman W, Weber R, Logan T, Buzoianu M (2010). Etaracizumab Melanoma Study Group: A randomized phase 2 study of etaracizumab, a monoclonal antibody against integrin α_v_β_3_, ± dacarbazine in patients with stage IV metastatic melanoma. Cancer.

[42] Reynolds AR, Hart IR, Watson AR, Welti JC, Silva RG, Robinson SD, Da Violante G, Gourlaouen M, Salih M, Jones MC (2009). Stimulation of tumor growth and angiogenesis by low concentrations of RGD-mimetic integrin inhibitors. Nat Med.

[43] Girotti MR, Saturno G, Lorigan P, Marais R (2014). No longer an untreatable disease: How targeted and immunotherapies have changed the management of melanoma patients. Mol Oncol.

